# A CHO-Expressed Pseudorabies Virus gD Subunit Vaccine Elicits Potent Neutralizing Antibodies and Confers Complete Protection Against Lethal Challenge in Mice

**DOI:** 10.3390/vaccines14080710

**Published:** 2026-08-18

**Authors:** Caoyuan Ma, Jia Li, Xin Song, Tao Wang, Qiang Yang, Ruojia Huang, Mengxiang Cao, Shengmei Chen, Yongfeng Li, Yuzi Luo, Yimin Wang, Lian-Feng Li, Hua-Ji Qiu, Hongxia Wu, Yuan Sun

**Affiliations:** 1 State Key Laboratory of Animal Disease Control and Prevention, Harbin Veterinary Research Institute, Chinese Academy of Agricultural Sciences, Harbin 150069, China; caoyuanma6@hotmail.com (C.M.); lijia0007070@163.com (J.L.); songxinor@163.com (X.S.); wangtao07@caas.cn (T.W.); 15045381827@163.com (Q.Y.); huangruojia2024@163.com (R.H.); caomengxiang13@163.com (M.C.); chenshengmei2023@163.com (S.C.); liyongfeng@caas.cn (Y.L.); luoyuzi@caas.cn (Y.L.); lilianfeng@caas.cn (L.-F.L.); qiuhuaji@caas.cn (H.-J.Q.); 2 Henan Institute of Science and Technology, Xinxiang 453003, China; yiminwang1@hotmail.com

**Keywords:** pseudorabies virus (PRV), glycoprotein D (gD), subunit vaccine, CHO cells, immunogenicity, neutralizing antibody

## Abstract

**Background/Objectives**: Pseudorabies virus (PRV) variant strains have caused widespread outbreaks in China since 2011, and currently available vaccines provide suboptimal protection. Glycoprotein D (gD), the principal target of virus-neutralizing antibodies, represents a promising antigen for subunit vaccine development. However, CHO cell-based production systems suitable for large-scale manufacturing remain insufficiently explored. This study aimed to develop a potentially scalable CHO cell-derived PRV gD subunit vaccine and evaluate its immunogenicity and protective efficacy in mice. **Methods:** A stable Chinese hamster ovary (CHO) suspension cell line secreting the extracellular domain of PRV gD was established through signal peptide optimization and stepwise serum-free adaptation. The recombinant gD protein was purified using Ni^2+^- Sepharose High-Performance affinity chromatography and subsequently formulated with MONTANIDE ISA 206 adjuvant. Immunogenicity and protective efficacy were assessed in BALB/c mice through serological analysis, neutralization assays, lethal challenge experiments, and quantitative PCR. **Results**: The gD subunit vaccine induced rapid seroconversion of gD-specific IgG antibodies as early as 7 days post immunization and exhibited a strong booster effect, maintaining high antibody levels. Neutralizing antibodies were first detected at 14 days and increased significantly after booster immunization, with titers markedly exceeding those induced by a commercial inactivated PRV vaccine at 42 days (*p* = 0.001). Following lethal challenge with 10^4^ TCID_50_ of the highly virulent PRV-TJ variant strain, vaccinated mice achieved 100% survival without clinical signs. Viral genome copy numbers in the brain and spinal cord were reduced by approximately 3.3 to 4.4 log_10_ relative to the PBS control group. **Conclusions**: The CHO cell-derived PRV gD subunit vaccine elicits robust humoral immune responses and provides complete protection against lethal PRV variant challenge in mice. These findings support its further evaluation in the natural swine host toward the development of a safe and scalable subunit vaccine for pseudorabies control.

## 1. Introduction

Pseudorabies (PR), also known as Aujeszky’s disease, is an acute and highly lethal infectious disease caused by pseudorabies virus (PRV), a member of the *Alphaherpesvirinae* subfamily within the *Herpesviridae* [1,2]. Pigs serve as the only natural host and the primary reservoir of PRV in the field, and PR outbreaks have caused substantial economic losses to the global swine industry [3]. The clinical manifestations of PRV infection vary with host age and physiological status: neonatal piglets exhibit severe neurological signs with mortality rates approaching 100%, growing–finishing pigs primarily display respiratory distress, and pregnant sows suffer from reproductive failure including abortion, stillbirth, and mummified fetuses [4]. Beyond swine, PRV can infect a broad range of mammals (including cattle, sheep, dogs, cats, and wild boar) and sporadic human cases have been documented since 2017, raising concerns about its zoonotic potential [5,6]. Critically, PRV establishes lifelong latent infection in the trigeminal ganglia and olfactory bulbs of surviving animals; the virus can be reactivated under stress or immunosuppression, leading to recurrent viral shedding and sustained transmission within herds [7].

Vaccination has been the cornerstone of PR control for decades. The live attenuated Bartha-K61 strain vaccine played a pivotal role in eradicating PR from domestic pig populations in several countries when combined with strict biosecurity measures [8]. However, since late 2011, highly virulent PRV variant strains have emerged in China and rapidly spread across vaccinated swine farms, causing severe and persistent outbreaks [9,10]. These variant strains exhibit enhanced virulence, increased neurotropism, and significant immune evasion capacity compared with classical strains, resulting in reduced efficacy of the Bartha-K61 vaccine [11,12]. Notably, genomic recombination between vaccine strains and field variant strains has been documented, raising further biosafety concerns regarding the continued use of live attenuated vaccines [13]. Consequently, the development of safer and more efficacious next-generation vaccines against circulating PRV variants has become an urgent research priority [14].

Among the PRV envelope glycoproteins, glycoprotein D (gD) is a type I transmembrane protein that serves as the key mediator of viral attachment and entry into host cells by interacting with cellular receptors including nectin-1, nectin-2, and HVEM [15,16]. Owing to its indispensable role in the viral entry process and its surface-exposed antigenic epitopes, gD is the principal target of virus-neutralizing antibodies and has been widely recognized as an ideal antigen for PR subunit vaccine development. Importantly, mutations in the gD extracellular domain of PRV variant strains have been shown to contribute to enhanced viral attachment, penetration efficiency, and neurotropism, further underscoring the antigenic significance of this glycoprotein [17]. A growing body of evidence demonstrates the protective efficacy of gD-based vaccines in preclinical models: gD subunit vaccines expressed in heterologous systems elicit durable neutralizing antibody responses and confer complete protection in piglets [18]. HEK-293T-expressed gD protein protected both mice and piglets against lethal challenge [19] and gD has been successfully incorporated into diverse platforms including DNA vaccines, mRNA-LNP vaccines, self-assembled nanoparticle vaccines, and Fc-fusion proteins [20,21,22,23,24]. These findings collectively validate gD as a robust and versatile target antigen for PR vaccine design.

The choice of expression system critically influences the yield, conformational authenticity, and immunogenicity of recombinant subunit vaccine antigens. Chinese hamster ovary (CHO) cells have become the predominant platform for industrial-scale production of therapeutic glycoproteins and subunit vaccines, accounting for over 70% of approved recombinant biologics [25]. In contrast to prokaryotic and insect cell expression systems, CHO cells facilitate complex post-translational modifications, including N-linked glycosylation, disulfide bond formation, and correct protein folding, thereby more closely approximating native mammalian glycoproteins and preserving conformational epitopes required for the induction of functional neutralizing antibodies [26,27]. A recent study further demonstrated that a CHO-K1-derived gB and gD subunit vaccine conferred complete clinical protection in piglets [28]. Nevertheless, two important gaps remain. First, the establishment of stable, high-producing CHO suspension cell lines adapted to serum-free culture, a prerequisite for cost-effective large-scale and cost-effective manufacturing, has not been systematically described for gD-only PRV subunit vaccines. Second, while previous gD vaccine studies have demonstrated protection in mice, comprehensive quantitative data on tissue-level viral clearance—particularly in the central nervous system (CNS), the primary target of PRV neuroinvasion—remain limited [29].

In the present study, we aimed to address these gaps by developing a CHO suspension cell-derived PRV gD subunit vaccine and evaluating its immunogenicity and protective efficacy in a mouse model. We first constructed a stable CHO cell line secreting the extracellular domain of PRV gD through systematic signal peptide optimization and stepwise serum-free adaptation. The recombinant gD protein was purified by affinity chromatography, formulated with the MONTANIDE ISA 206 adjuvant, and evaluated in BALB/c mice for its ability to induce gD-specific IgG and virus-neutralizing antibodies, confer protection against lethal challenge with the highly virulent PRV-TJ variant strain, and suppress viral replication in peripheral tissues. Collectively, our results demonstrate that the CHO-expressed gD subunit vaccine elicits robust humoral immune responses and confers complete protection against lethal challenge with a PRV variant, thereby supporting its further evaluation in the natural host toward the development of an efficacious subunit vaccine candidate.

## 2. Materials and Methods

### 2.1. Cells and Viruses

Chinese hamster ovary K1 (CHO-K1) cells were cultured in Kaighn’s modified Ham’s F-12 medium (Ham’s F-12K; Procell, Wuhan, China, PM150910) supplemented with 10% fetal bovine serum (FBS; Gibco, Grand Island, NY, USA) and 1% antibiotic–antimycotic solution (Gibco, Grand Island, NY, USA, 15140122). Following stable transfection, CHO-K1 cells were gradually adapted to suspension culture and maintained in serum-free CHO Grow CD2 medium (BasalMedia, Shanghai, China, H120KJ) supplemented with 1% L-glutamine (BasalMedia, Shanghai, China, S210V) at 37 °C in a humidified atmosphere containing 5% CO_2_ with continuous shaking at 115 rpm. Cell density and viability were monitored daily using an automated cell counter (Shanghai RuiYu Biotech Co., Ltd., Shanghai, China, IC1000).

PK-15 cells were maintained in Dulbecco’s Modified Eagle Medium (DMEM; Gibco, Grand Island, NY, USA, C11995500BT) supplemented with 10% FBS and 1% antibiotic–antimycotic solution at 37 °C in a humidified incubator containing 5% CO_2_.

The highly virulent PRV variant strain TJ (PRV-TJ; GenBank accession no. KJ789182.1), originally isolated in Tianjin, China, was used for all challenge experiments [30]. Viral titers were determined on PK-15 cells and expressed as the 50% tissue culture infective dose (TCID_50_) using the Reed–Muench method.

### 2.2. Animals and Ethics Statement

Female BALB/c mice (6 weeks old) were purchased from Liaoning Changsheng Biotechnology Co., Ltd. (Shenyang, China). Animals were housed under specific pathogen-free (SPF) conditions with ad libitum access to food and water throughout the study.

All animal experiments were approved by the Animal Ethics Committee of Harbin Veterinary Research Institute (HVRI), Chinese Academy of Agricultural Sciences (CAAS), China (approval no. HVRI-250306-01-GR), and were conducted in accordance with the relevant institutional guidelines and regulations for animal welfare.

Mice were anesthetized with 1.5% isoflurane (flow rate: 1 L/min) during immunization and challenge procedures. At the experimental endpoint, animals were humanely euthanized by cervical dislocation in accordance with the approved ethical protocols.

### 2.3. Construction of the gD Expression Vector

The transmembrane domain of the PRV *gD* gene was predicted using TMHMM 2.0 and removed to facilitate secretion of the recombinant protein. The extracellular region of gD was fused at the N-terminus with one of three signal peptides, including interleukin 2 (IL-2), glycosyltransferase family 6 (GT6), or azurocidin preproprotein (AP), to evaluate secretion efficiency in mammalian cells. Strep and 6×His affinity tags were appended to the C-terminus to enable protein purification and detection. The codon-optimized *gD* gene for expression in Chinese hamster ovary (CHO) cells was synthesized by BGI Genomics (Shenzhen, China) with EcoRI and XhoI (Thermo Fisher Scientific, Waltham, MA, USA) restriction sites. The gene fragments were inserted into pcDNA3.1(+) and pCAG-3×FLAG-IRES-Neo expression vectors using the ClonExpress II One Step Cloning Kit (Vazyme, Nanjing, China, C112-01) according to the manufacturer’s instructions. Recombinant expression vectors were transformed into Escherichia coli DH5*α* competent cells and cultured in Luria–Bertani (LB) medium containing ampicillin (50 μg/mL; Sigma-Aldrich, St. Louis, MO, USA, A9518). Positive clones were verified by sequencing analysis, and endotoxin-free plasmid DNA was prepared using an EndoFree Maxi Plasmid Kit (Tiangen, Beijing, China, DP117) for subsequent transfection experiments. Additional expression constructs were generated using the same homologous recombination strategy.

### 2.4. Generation of Stable CHO Suspension Cell Line

To determine the optimal construct for stable expression, the expression vectors described above were individually transfected into CHO-K1 cells, followed by preliminary assessment of recombinant protein expression by Western blot. The pcDNA-IL2-gD construct demonstrated consistently higher gD expression than the other variants and was therefore selected for subsequent stable cell line establishment.

To establish stable gD-expressing cell lines, CHO-K1 cells (10^5^ cells) were transfected with 10 μg of the pcDNA-IL2-gD expression vector by electroporation using EBEL transfection buffer (Etta Biotech Co., Ltd., Suzhou, China, H10305). Electroporation was performed at 300 V with a pulse width of 5 ms for six pulses at 100 ms intervals according to the manufacturer’s recommendations. Transfected cells were cultured for 48 h prior to antibiotic selection. Stable transfectants were selected using G418 (800 μg/mL; Sigma-Aldrich, St. Louis, MO, USA, A1720). Surviving cells were subjected to single-cell cloning by limiting dilution in 96-well plates to obtain monoclonal cell populations. Positive clones were screened for recombinant gD expression by Western blot.

Selected clones were subsequently adapted to serum-free suspension culture through stepwise replacement with CHO Grow CD2 medium (BasalMedia, Shanghai, China, H120KJ). After complete adaptation, cells were maintained in a shaking incubator as described in Section 2.1. Recombinant protein expression stability was monitored by Western blot analysis over 20 serial passages. Established stable CHO-gD suspension cells were cryopreserved in liquid nitrogen using freezing medium supplemented with 10% dimethyl sulfoxide (DMSO; Sigma-Aldrich, St. Louis, MO, USA, D2650) for long-term storage.

### 2.5. Expression and Purification of the Recombinant gD Protein

Stable CHO-gD suspension cells were cultured in 300 mL of serum-free CHO Grow CD2 medium at an initial density of 10^6^ cells/mL under shaking conditions, as described in Section 2.1. Culture supernatants were harvested when recombinant gD protein expression reached peak levels (day 5 post-seeding). The cultures were centrifuged at 8000× *g* for 10 min at 4 °C to remove cell debris, and the supernatants were further clarified by filtration through a 0.45 μm Millex-HV filter (Millipore, Billerica, MA, USA, SLHV033RS).

Clarified supernatants were loaded onto a Ni^2+^-Sepharose High Performance affinity chromatography column (Cytiva, Marlborough, MA, USA, 17526801) pre-equilibrated with 50 mM phosphate-buffered saline (PBS, pH 7.3) at a flow rate of 1 mL/min. Bound recombinant gD protein was eluted using a stepwise imidazole gradient (50 mM, 100 mM, and 300 mM imidazole in PBS; five column volumes per step), and fractions corresponding to the 300 mM imidazole elution peak were collected. The eluted protein was desalted by dialysis against PBS and subsequently concentrated. Protein purity was evaluated by sodium dodecyl sulfate-polyacrylamide gel electrophoresis (SDS-PAGE) followed by Coomassie Brilliant Blue staining and by Western blot analysis using an anti-His monoclonal antibody. Protein concentration was determined using a bicinchoninic acid (BCA) protein assay kit (Solarbio, Beijing, China, PC0020). The purified recombinant gD protein was aliquoted and stored at −80 °C until further use.

### 2.6. Vaccine Formulation

The purified recombinant gD protein was emulsified with MONTANIDE ISA 206 VG adjuvant (Seppic, Paris, France), a water-in-oil-in-water mineral oil-based emulsion, at a 1:1 (*v*/*v*) ratio according to the manufacturer’s instructions to obtain a homogeneous vaccine formulation. Each dose for mouse immunization contained 20 μg of recombinant gD protein in a final volume appropriate for intramuscular administration.

Control formulations were prepared as follows: the adjuvant-only group received PBS emulsified with MONTANIDE ISA 206 VG at a 1:1 (*v*/*v*) ratio; the negative control group received sterile PBS alone; and the positive control group received a commercially available inactivated PRV vaccine (inactivated PRV Ea strain; China Animal Husbandry Corporation, Chengdu, China). The commercial inactivated PRV vaccine was supplied at one dose per 2 mL, and each mouse received 0.1 dose (200 μL) per immunization. The immunization schedule was the same as that for the gD vaccine group, i.e., prime on day 0 and boost on day 21. All vaccine formulations were prepared under sterile conditions and administered immediately after preparation.

### 2.7. Mouse Immunization, Viral Challenge, and Protection Evaluation

#### 2.7.1. Immunization and Experimental Design

A total of thirty-two 6-week-old female BALB/c mice were randomly assigned to four groups (*n* = 8 per group) using a complete randomization method: (Group A) gD subunit vaccine group (20 μg recombinant gD protein emulsified with MONTANIDE ISA 206 VG adjuvant); (Group B) positive control group (commercial inactivated PRV vaccine); (Group C) adjuvant control group (PBS emulsified with MONTANIDE ISA 206 VG); and (Group D) negative control group (PBS alone). All mice were immunized intramuscularly in the quadriceps of the hind limb on days 0 and 21, with a 21-day interval between immunizations. Of the eight mice in each group, five were randomly designated for immunogenicity evaluation (serum antibody detection) and survival analysis following viral challenge, and the remaining three were used for viral load determination in major tissues (brain, muscle, spinal cord, and kidney) collected at 3 days post challenge (dpc).

Peripheral blood samples were collected via the retro-orbital plexus at 0, 7, 14, 21, 28, 35, and 42 days post immunization (dpi). Blood samples were allowed to stand at room temperature for 30 min, and sera were separated by centrifugation at 3000× *g* for 10 min, aliquoted, and stored at −20 °C until further analysis.

#### 2.7.2. Challenge Dose Determination

The challenge dose was selected based on previously established challenge conditions for PRV-TJ infection in BALB/c mice [7]. Mice were challenged intramuscularly with 10^4^ TCID_50_ of PRV-TJ.

#### 2.7.3. Viral Challenge and Clinical Monitoring

At 42 dpi, all mice were challenged intramuscularly with 10^4^ TCID_50_ of PRV-TJ in a 100 μL volume. Following challenge, mice were monitored daily for 10 days for clinical signs, body weight changes, and survival. Clinical symptoms were scored according to the following criteria: 0, no clinical signs; 1, pruritus at the inoculation site with scratching and biting; 2, restlessness and ruffled fur; 3, self-mutilation, skin lesions at the inoculation site, and depression; 4, moribund or dead. Mice reaching a clinical score of 4 or exhibiting severe neurological signs (e.g., persistent tremors, ataxia, or inability to access food and water) were euthanized immediately in accordance with institutional animal welfare guidelines and recorded as non-survivors for the subsequent time point. Body weight and survival were recorded daily throughout the 10-day observation period.

#### 2.7.4. Tissue Collection

To assess viral replication in target tissues, the three mice pre-designated for tissue collection in each group were euthanized at 3 dpc (corresponding to the onset of mortality in the PBS control group). Brain, spinal cord, hindlimb skeletal muscle (*Quadriceps femoris*), and kidney tissues were collected aseptically, snap-frozen in liquid nitrogen, and stored at −80 °C until viral load quantification (Section 2.8).

### 2.8. Evaluation of Humoral Immune Responses

#### 2.8.1. gD-Specific Antibody Detection

The assay was performed using an indirect enzyme-linked immunosorbent assay (ELISA) as described previously [23], with minor modifications. Briefly, 96-well ELISA plates (Corning, New York, NY, USA, 3590) were coated with 100 μL/well of purified recombinant gD protein (1 μg/mL in coating buffer) overnight at 4 °C. Plates were washed three times with PBST (PBS containing 0.05% Tween-20) and blocked with 2% bovine serum albumin (BSA; Sigma-Aldrich, St. Louis, MO, USA, A7906) for 2 h at room temperature. Serum samples (1:1000 in blocking buffer, 100 μL/well) were added and incubated for 90 min at 37 °C. Following three PBST washes, HRP-conjugated goat anti-mouse IgG (1:10000; Beyotime, Shanghai, China, A0216) was added (100 μL/well) and incubated for 45 min at 37 °C. Plates were washed again, and 100 μL/well of TMB substrate solution (Solarbio, Beijing, China, PR1200) was added in the dark at room temperature for approximately 30 min, until the OD_450_ of the positive control reached 1.5–2.0. The reaction was stopped with 50 μL of 2 M H_2_SO_4_, and absorbance was measured at 450 nm using a microplate reader (BioTek, Winooski, VT, USA, ELx800).

#### 2.8.2. Virus Neutralization Assay

Serum neutralizing antibody (NAb) titers were determined using a serum–virus neutralization assay on PK-15 cells, as described previously [10], with minor modifications. All serum samples were heat-inactivated at 56 °C for 30 min and serially 2-fold diluted (1:4 to 1:512) in DMEM containing 2% FBS. Each dilution was mixed with an equal volume of PRV-TJ virus suspension (200 TCID_50_) and incubated at 37 °C for 1 h to enable neutralization. The mixture was then added to 96-well plates containing ~80% confluent PK-15 cell monolayers. After a 72 h incubation at 37 °C in a humidified 5% CO_2_ atmosphere, cytopathic effect (CPE) was observed under an inverted microscope.

The NAb titer was defined as the reciprocal of the highest serum dilution that inhibited ≥50% CPE relative to virus-only control wells, calculated via the Reed–Muench method [30]. A geometric mean titer (GMT) of ≥1:16 was used as a previously reported reference threshold associated with protective neutralizing antibody responses in PRV vaccine studies [19].

### 2.9. Tissue Viral Load Determination

Viral DNA loads in tissues of challenged mice were quantified by quantitative real-time PCR (qPCR). Brain, spinal cord, skeletal muscle, and kidney tissues collected at 3 dpc (described in Section 2.7.4) were homogenized in PBS using a tissue homogenizer (Tiangen, Beijing, China). Total genomic DNA was extracted using the TGuide Smart Universal Genomic DNA Extraction Kit (Tiangen, Beijing, China, DP605) on the TGuide S32 Automated Nucleic Acid Extraction and Purification System (Tiangen, Beijing, China, YOSE-S32) according to the manufacturer’s instructions. The concentration and purity of extracted DNA were assessed spectrophotometrically by measuring A260/A280 and A260/A230 ratios. Samples with A260/A280 ratios between 1.8 and 2.0 were considered acceptable for downstream analysis.

Viral genome copy numbers were quantified by qPCR targeting the PRV *gB* gene, using primers and reaction conditions as described previously [31]. A standard curve was generated using 10-fold serial dilutions of a plasmid standard containing the PRV *gB* gene fragment of known copy number. Viral loads were normalized to tissue weight and expressed as viral genome copy numbers per milligram of tissue.

### 2.10. Statistical Analysis

All statistical analyses were performed using GraphPad Prism version 10 (GraphPad Software Inc., La Jolla, CA, USA). Data are presented as the mean ± SD. Statistical significance was defined as follows: * *p* < 0.05, ** *p* < 0.01, *** *p* < 0.001, and **** *p* < 0.0001; ns, not significant (*p* ≥ 0.05).

Serum gD-specific IgG levels (OD_450_) and neutralizing antibody titers (log_2_-transformed reciprocal titers) at 0, 7, 14, 21, 28, 35, and 42 dpi were analyzed by two-way ANOVA (group × time) followed by Dunnett’s test, with the gD subunit vaccine group serving as the reference against which the commercial inactivated PRV vaccine, adjuvant control, and PBS control groups were compared. Body weight changes after challenge, expressed as the percentage of body weight 1 day before challenge, and clinical scores were analyzed by two-way repeated-measures ANOVA (group × time) with Geisser–Greenhouse correction and Tukey’s test; pairwise comparisons at each post-challenge time point compared the gD vaccine group with each of the other three groups. Survival curves were compared using the log-rank (Mantel–Cox) test, and pairwise comparisons between the gD subunit vaccine group and the other three groups were performed with Bonferroni correction. Viral genome copy numbers in brain, spinal cord, skeletal muscle, and kidney at 3 days post-challenge were analyzed for each tissue separately by one-way ANOVA with Dunnett’s test, again using the gD subunit vaccine group as the reference.

For each experimental group (*n* = 8), five mice were used for immunogenicity assessment (serum antibody detection) and survival analysis, and the remaining three mice were euthanized at 3 days post challenge for tissue viral load determination.

## 3. Results

### 3.1. Construction and Characterization of the Recombinant gD Vector

**Figure 1:**
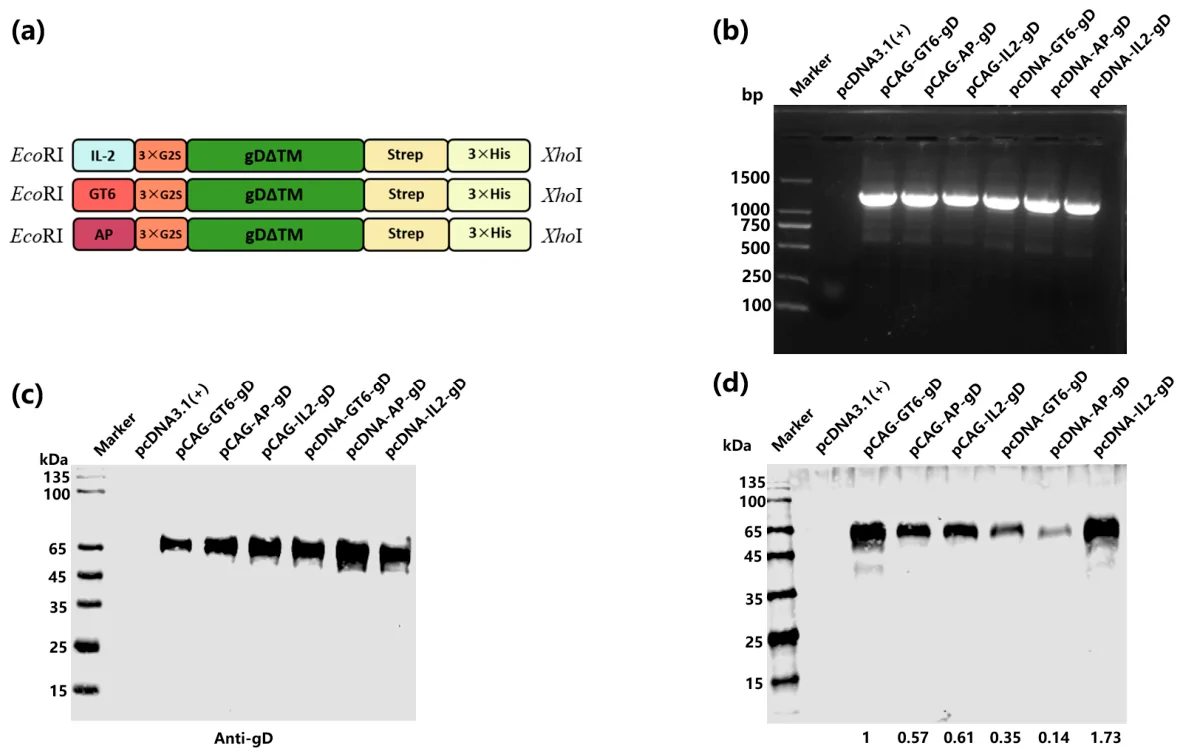
Construction and expression analysis of recombinant PRV gD in CHO cells. (**a**) Schematic diagram of the PRV glycoprotein D (gD) expression constructs, in which the gD open reading frame was fused to three different signal peptides (IL-2, GT6, and AP) and tagged with Strep and 6×His epitopes. (**b**) Restriction enzyme digestion analysis using EcoRI and XhoI confirming insertion of the *gD* gene into the pcDNA3.1(+) and pCAG-3×FLAG-IRES-Neo expression vectors. (**c**) Western blot analysis of gD expression in CHO-K1 cells following transient transfection, detected using an anti-His antibody. (**d**) Western blot detection of secreted gD protein in cell culture supernatants collected at 72 h post electroporation from CHO cells expressing gD with different signal peptides.

The recombinant gD protein was designed to include N-terminal signal peptides (IL-2, GT6, or AP) and two C-terminal tags (Strep and 6×His). The codon-optimized sequence (for CHO cell expression) was inserted into the pcDNA3.1(+) and pCAG-3×FLAG-IRES-Neo vectors via the EcoRI and XhoI restriction sites, yielding the recombinant plasmids pcDNA-IL2-gD, pcDNA-GT6-gD, pcDNA-AP-gD, pCAG-IL2-gD, pCAG-GT6-gD, and pCAG-AP-gD (Figure 1a). Restriction enzyme digestion produced fragments consistent with the expected size (Figure 1b). All six constructs were individually transfected into CHO-K1 cells to evaluate transient gD expression. Western blot of cell lysates using an anti-His antibody detected a specific band of approximately 55 kDa, corresponding to the expected molecular weight of recombinant gD (Figure 1c). To compare secretion efficiency among the different signal peptides, culture supernatants were collected at 72 h post-electroporation, concentrated using Ni^2+^ affinity resin, and analyzed by Western blot. Among the constructs tested, pcDNA-IL2-gD yielded the highest level of secreted gD protein and was therefore selected for subsequent stable cell line generation (Figure 1d).

### 3.2. gD Sequence Conservation Among PRV Strains

**Figure 2:**
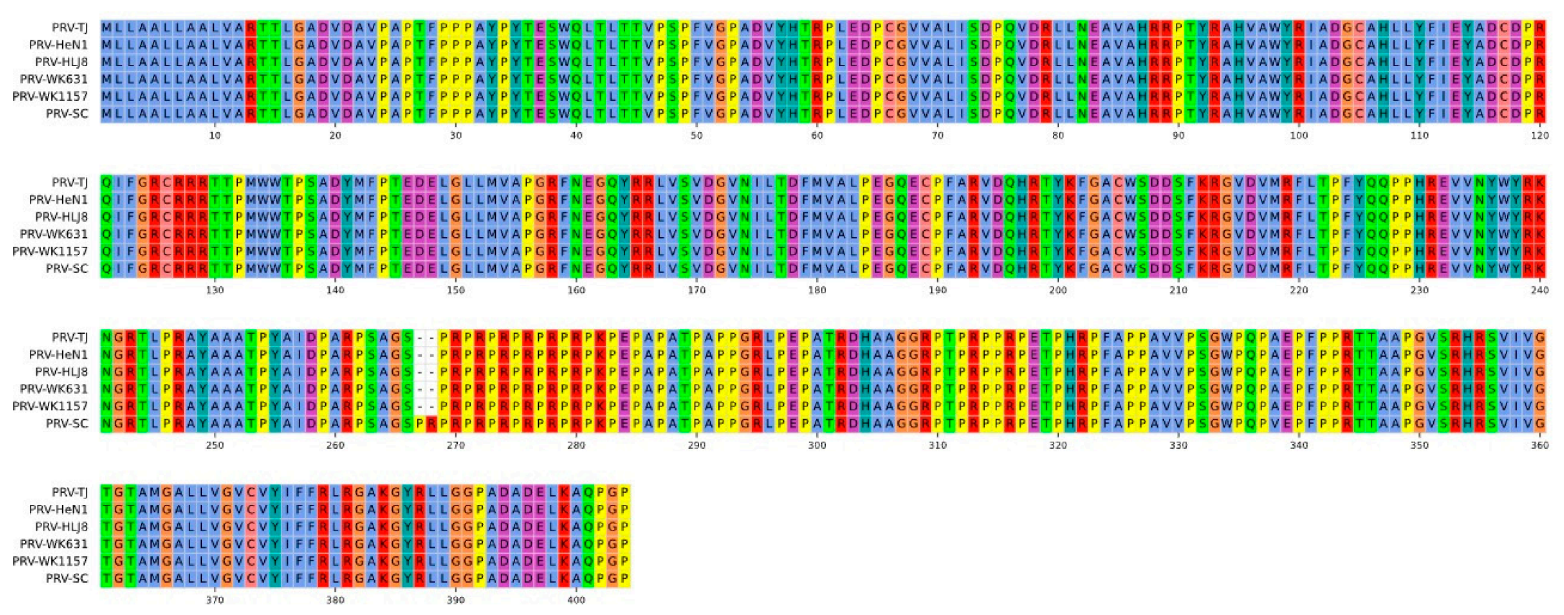
Amino acid sequence alignment of the PRV gD extracellular domain among representative pseudorabies virus strains. The gD amino acid sequence of the PRV-TJ strain (GenBank accession no. KJ789182.1), which served as the template for the recombinant gD expression construct in this study, was aligned with those of four PRV variant strains currently prevalent in China—HeN1 (GenBank accession no. KP098534.1), HLJ8 (GenBank accession no. KT824771.1), WK1157 (GenBank accession no. PX439454.1), and WK631 (GenBank accession no. PX439453.1)—and the classical PRV SC strain (GenBank accession no. KT809429.1). Multiple sequence alignment was performed using the MUSCLE algorithm via the Novopro online tool (www.novopro.cn/tools/muscle.html, accessed on 7 August 2026).

To evaluate the degree of amino acid sequence conservation of the gD extracellular domain among circulating PRV strains, we performed multiple sequence alignment of the gD sequence from the PRV-TJ strain (used as the template for our recombinant construct) with those of representative PRV variant strains currently prevalent in China [HeN1 (GenBank accession no. KP098534.1), HLJ8 (GenBank accession no. KT824771.1), WK1157 (GenBank accession no. PX439454.1), and WK631 (GenBank accession no. PX439453.1)] and the classical SC strain (GenBank accession no. KT809429.1), using the multiple sequence alignment tool provided by Novopro [www.novopro.cn/tools/muscle.html (accessed on 7 August 2026)]. The gD amino acid sequence of the TJ strain shared 100% identity with all four variant strains tested and 99.75% identity with the classical SC strain, with only a single amino acid substitution distinguishing TJ from SC (Figure 2). These results demonstrate that the gD extracellular domain is highly conserved among the PRV strains analyzed in this study, supporting the use of the TJ strain-derived gD sequence as a representative antigen for further vaccine evaluation.

### 3.3. Establishment of Stable Suspension Cell Line Expressing gD Protein

**Figure 3:**
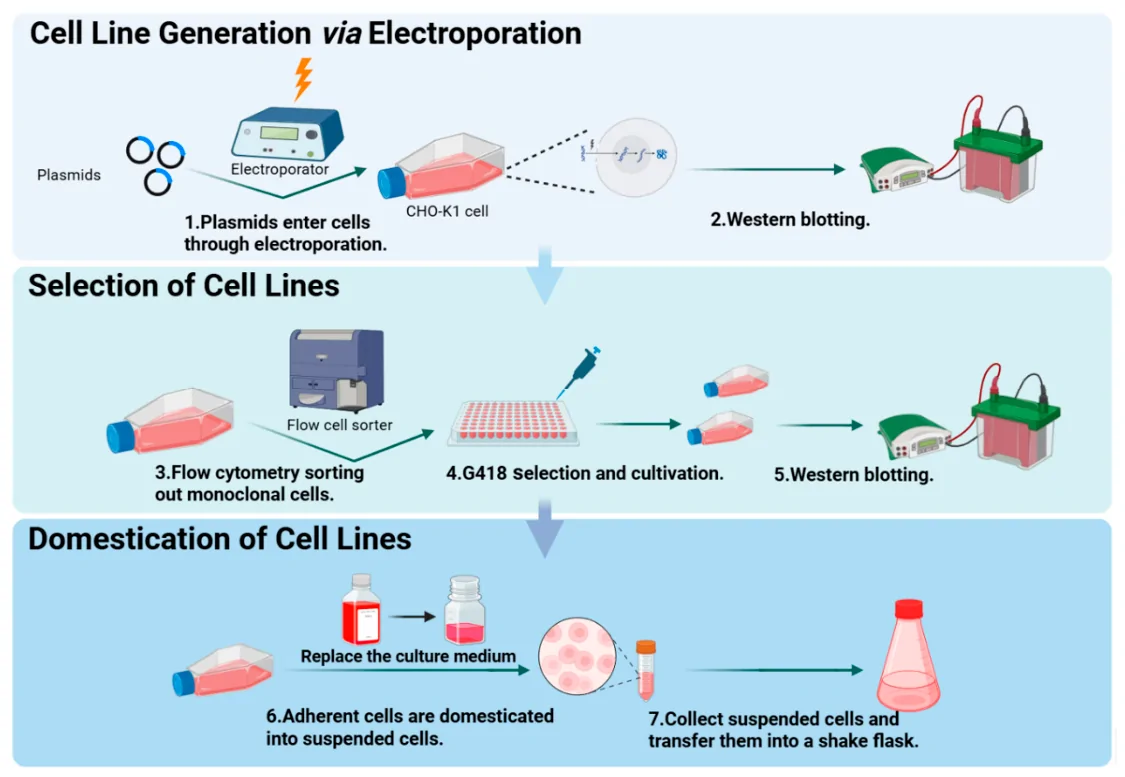
Schematic diagram of constructing stable CHO cell lines.

Plasmids were first transfected into adherent CHO-K1 cells via electroporation, and target protein expression was verified by Western blot. To isolate stable monoclonal cell lines, the transfected cells were serially diluted and subjected to G418 selection pressure before subsequent expansion. Finally, the adherent lines were adapted to serum-free medium through a stepwise serum reduction protocol to successfully establish a stable CHO suspension cell line secreting the gD protein (Figure 3).

**Figure 4:**
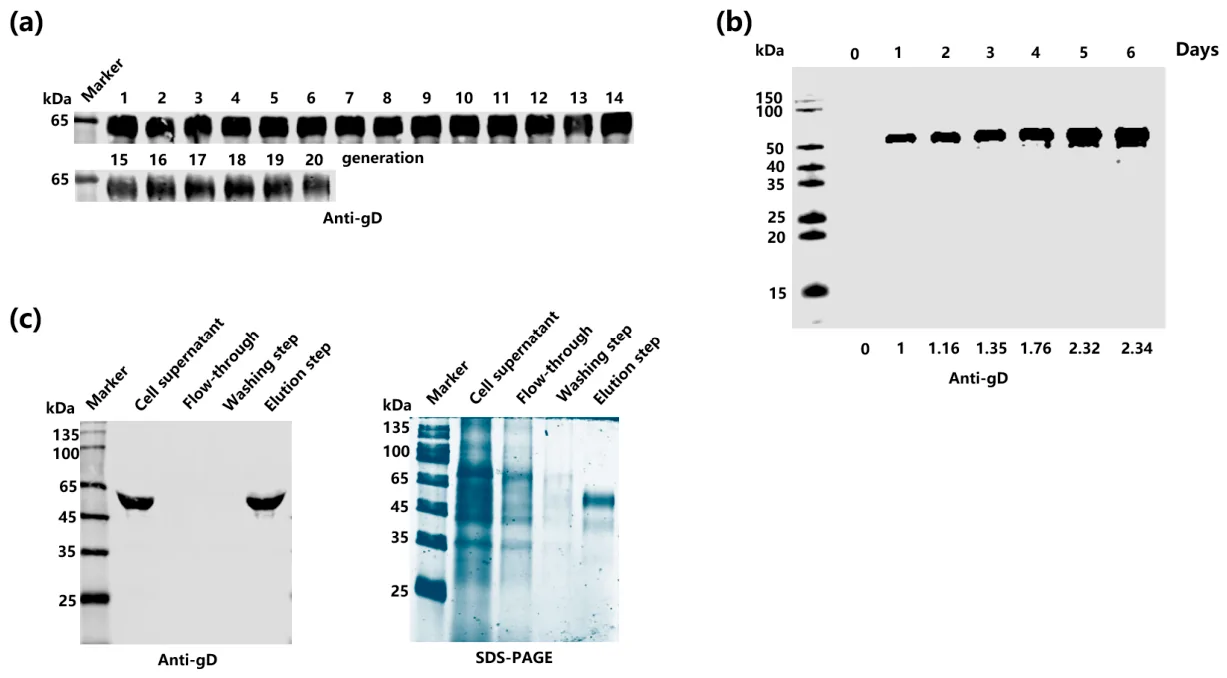
Establishment and characterization of a stable gD-expressing CHO suspension cell line. (**a**) Western blot analysis of gD expression across 20 consecutive passages of the stable CHO cell line, demonstrating expression stability. (**b**) Time-course analysis of gD protein secretion in culture supernatants from day 0 to day 6, together with corresponding cell density and cell viability measurements. (**c**) Purification and characterization of the recombinant gD protein, analyzed by Western blot (**left panel**) and SDS-PAGE (**right panel**).

Western blot analysis of culture supernatants collected over 20 consecutive passages confirmed that the established stable CHO-gD cell line maintained consistent gD protein secretion throughout the passage period. The purified recombinant gD protein yield was 150 ± 10 μg per mL of culture supernatant, as quantified by BCA protein assay from 300 mL shake-flask cultures (Figure 4a).

A time-course analysis of gD secretion over an 8-day batch culture revealed that gD protein progressively accumulated in the supernatant, reaching a maximum concentration on day 5, after which the level plateaued. Concurrent monitoring of cell density and viability demonstrated that the culture maintained high viability through day 5, with a gradual decline thereafter, consistent with the observed plateau in protein secretion (Figure 4b). The recombinant gD protein was purified from culture supernatants by Ni^2+^- Sepharose High Performance affinity chromatography. SDS-PAGE analysis of the purified product showed a single predominant band at approximately 55 kDa, and Western blot with an anti-His antibody confirmed the identity of the purified protein (Figure 4c).

### 3.4. Immune Responses to gD Subunit Vaccine in Mice

**Figure 5:**
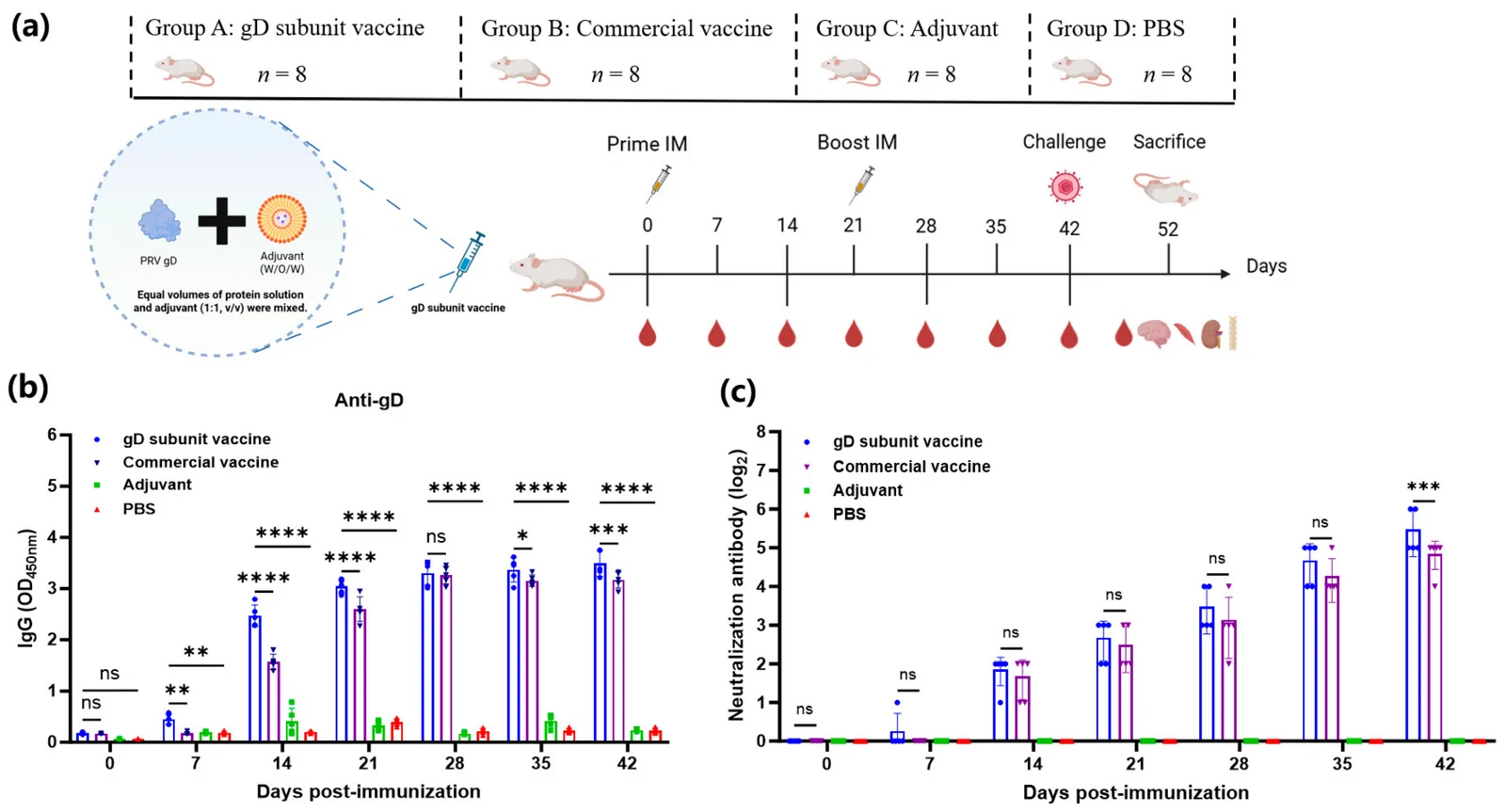
Humoral immune responses induced by the gD subunit vaccine in mice. (**a**) Immunization and challenge schedule for BALB/c mice. (**b**) gD-specific IgG antibody levels in mouse sera at the indicated time points following immunization. Data are presented as mean ± SD (*n* = 5 per group). Statistical analysis was performed using two-way ANOVA (factors: group × time) followed by Dunnett’s multiple comparisons test, with the gD subunit vaccine group set as the reference control against which the commercial inactivated PRV vaccine, adjuvant, and PBS groups were compared at each time point. * *p* < 0.05, ** *p* < 0.01, *** *p* < 0.001, and **** *p* < 0.0001 indicate significant differences versus the gD subunit vaccine group; ns, not significant (*p* ≥ 0.05). Exact *p*-values are reported in the main text. (**c**) Virus neutralizing antibody (NAb) titers in mouse sera. Data are presented as GMTs (*n* = 5 per group). Statistical analysis was performed as described in (**b**).

BALB/c mice were immunized as described in Section 2.7 with the gD subunit vaccine, commercial inactivated PRV vaccine (positive control), adjuvant alone, or PBS (negative control) on days 0 and 21 (*n* = 5 per group for immunogenicity analysis). Serum samples were collected at 0, 7, 14, 21, 28, 35, and 42 dpi for antibody analysis (Figure 5a).

Indirect ELISA against recombinant gD protein revealed that gD-specific IgG antibodies became detectable as early as 7 dpi in the gD vaccine group. Following the booster immunization at 21 dpi, antibody levels increased markedly and were maintained at elevated levels through 42 dpi. gD-specific IgG levels in the gD vaccine group were significantly higher than those in the adjuvant and PBS control groups from 7 dpi onward (day 7: *p* = 0.0072 and *p* = 0.0058, respectively; days 14–42: all *p* < 0.0001). Compared with the commercial inactivated PRV vaccine group, gD-specific IgG levels were significantly higher at 7 dpi (*p* = 0.0041), 14 dpi (*p* < 0.0001), and 21 dpi (*p* < 0.0001); were comparable at 28 dpi (*p* = 0.9360, 7 days after the booster dose); and were again significantly higher at 35 dpi (*p* = 0.0390) and 42 dpi (*p* = 0.0008) (Figure 5b).

Virus NAb titers, determined by the serum–virus neutralization assay on PK-15 cells, became detectable at 14 dpi (GMT ≈ 1:8) and were substantially enhanced after the booster dose. At 28 dpi, the geometric mean titer (GMT) in the gD vaccine group reached 1:32, significantly exceeding those of the adjuvant and PBS control groups (*p* < 0.001), and was comparable to the GMT of the commercial inactivated PRV vaccine group. At 42 dpi, the NAb GMT in the gD vaccine group rose further to 1:45, significantly surpassing that of the commercial vaccine group (*p* = 0.001), and far exceeding the established protective threshold of GMT ≥ 1:16 [19]. At a serum dilution of 1:32, the gD vaccine group achieved a virus inhibition rate of 89.7% ± 3.2%. At 35 dpi (14 days post-boost), the GMT remained at 1:28, indicating that the vaccine induced a durable neutralizing antibody response (Figure 5c).

### 3.5. Protection of Mice from Lethal PRV Challenge by gD Subunit Vaccine

To evaluate protective efficacy, immunized mice were challenged intramuscularly with 10^4^ TCID_50_ of the highly virulent PRV-TJ variant strain at 42 dpi and monitored for 10 days.

**Figure 6:**
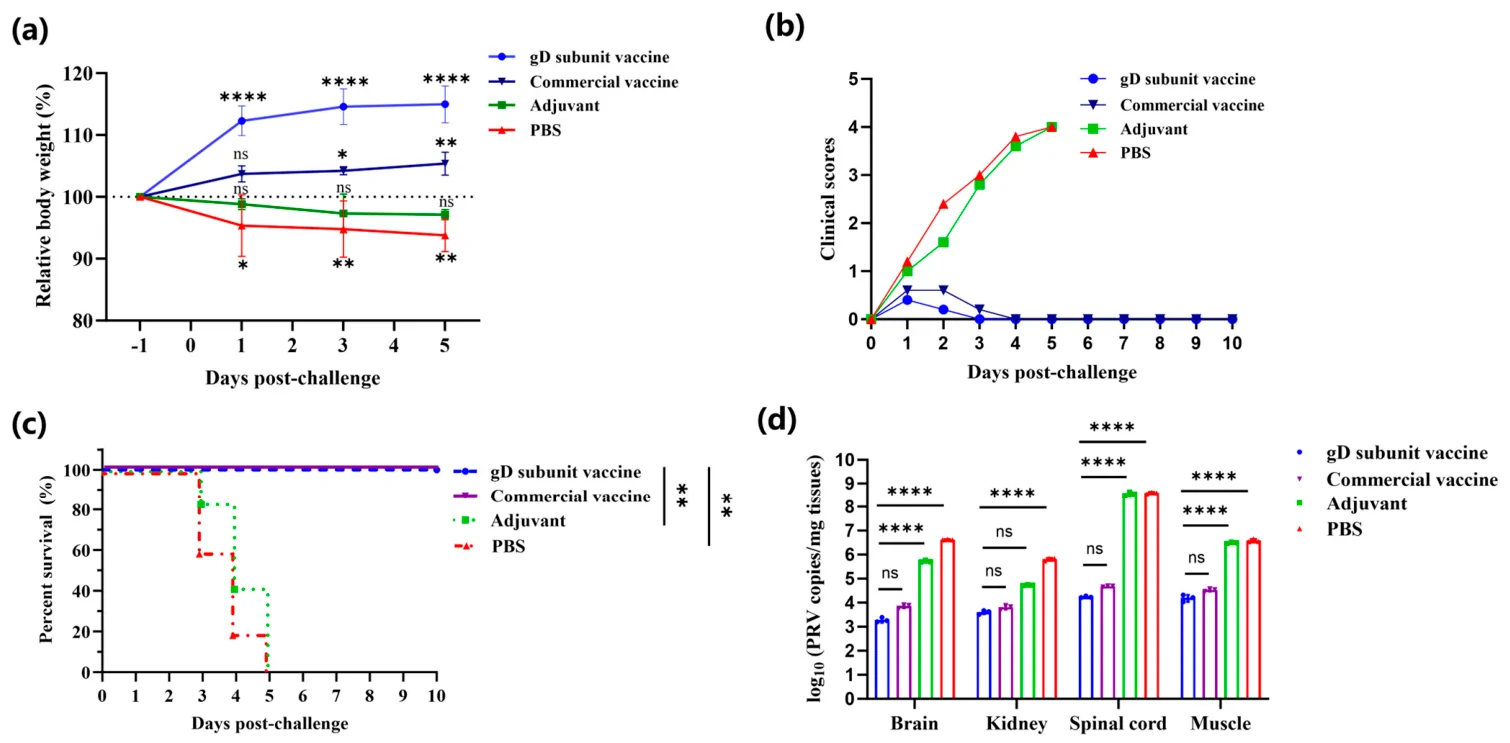
The protective efficacy of the gD subunit vaccine against lethal PRV challenge in mice. (**a**) Relative body weight changes following challenge with 10^4^ TCID_50_ of PRV-TJ. Body weights are expressed as percentages of body weight measured 1 day before challenge. Data are presented as the mean ± SD (*n* = 5 per group). (**b**) Clinical scores of immunized mice following PRV challenge. Data are presented as mean ± SD (*n* = 5 per group). Statistical analysis was performed as described in (**a**). (**c**) Survival curves. Survival was monitored daily for 10 days post-challenge (*n* = 5 per group). Exact *p*-values for pairwise comparisons with the gD subunit vaccine group are indicated in the text. (**d**) Viral genome copy numbers in brain, spinal cord, skeletal muscle, and kidney tissues collected at 3 days post challenge. Data are presented as the mean ± SD (*n* = 3 per group). For all panels: * *p* < 0.05, ** *p* < 0.01, *** *p* < 0.001, and **** *p* < 0.0001; ns, not significant (*p* ≥ 0.05).

Mice immunized with either the gD subunit vaccine or the commercial inactivated PRV vaccine maintained or gained body weight throughout the observation period after challenge, whereas mice in the adjuvant-only and PBS control groups exhibited progressive weight loss (Figure 6a). In the gD-vaccinated group, body weight remained significantly higher than the pre-challenge baseline at all post-challenge time points (*p* < 0.001). A similar trend was observed in the commercial vaccine group, although the increase reached statistical significance only at the later time points. In contrast, body weight remained relatively stable in the adjuvant-only group but declined significantly in the PBS control group following PRV challenge. A significant group × time interaction was observed (*p* < 0.0001).

Consistent with the body weight results, mice vaccinated with either the gD subunit vaccine or the commercial inactivated PRV vaccine maintained clinical scores of zero throughout the study and exhibited no observable signs of PRV infection. In contrast, mice in the adjuvant-only and PBS control groups rapidly developed severe clinical manifestations, including pruritus, ruffled fur, self-mutilation, and depression, and subsequently became moribund or died (Figure 6b).

Survival analysis further demonstrated that the gD subunit vaccine conferred complete protection against lethal PRV challenge, with all vaccinated mice surviving the 10-day observation period (100% survival), comparable to the commercial inactivated vaccine group (Figure 6c). In contrast, all mice in the adjuvant-only and PBS control groups succumbed to infection within 7 days. Survival was significantly improved in the gD-vaccinated group compared with the adjuvant-only and PBS control groups (*p* = 0.0026 and *p* = 0.0019, respectively).

To evaluate vaccine-mediated suppression of viral replication, viral genome copy numbers were quantified by qPCR in the brain, spinal cord, skeletal muscle, and kidney at 3 days post-challenge (*n* = 3 per group). High viral genome copy numbers were detected in all tissues from the PBS and adjuvant control groups, whereas viral genome copy numbers were markedly reduced in both vaccine groups (Figure 6d). In the brain, viral genome copy numbers in the gD-vaccinated group were reduced by approximately 3.3 log_10_ relative to the PBS control group (*p* < 0.0001). An even greater reduction of approximately 4.4 log_10_ was observed in the spinal cord, the principal target of PRV neuroinvasion. Viral genome copy numbers were also reduced by approximately 2.4 log_10_ in skeletal muscle and 2.2 log_10_ in the kidney (all *p* < 0.0001 versus both control groups). No significant differences were detected between the gD vaccine and commercial inactivated vaccine groups in any of the tissues examined (brain, *p* = 0.9994; spinal cord, *p* > 0.9999; skeletal muscle, *p* = 0.9998; kidney, *p* = 0.9997).

Consistent with these virological findings, no neurological signs, including tremors or ataxia, were observed in mice immunized with either the gD subunit vaccine or the commercial inactivated PRV vaccine throughout the observation period.

## 4. Discussion

In the present study, we systematically evaluated the effects of different promoters and signal peptides on the expression of recombinant PRV gD protein in CHO cells. Among the six vector–signal peptide combinations tested (pcDNA3.1 and pCAG vectors paired with IL-2, GT6, or AP signal peptides), the pcDNA-IL2-gD construct consistently produced the highest level of recombinant gD protein, as determined by Western blot analysis, and was therefore selected for the establishment of a stable CHO cell line. This superior performance was likely attributable to the combined effects of promoter activity and signal peptide efficiency. The pcDNA3.1 vector contains the human cytomegalovirus (CMV) immediate–early promoter, whereas the pCAG vector incorporates the CMV enhancer together with the chicken *β*-actin promoter. Although the CAG promoter has been widely used to achieve robust transgene expression in various mammalian cell types, promoter activity is known to be cell type- and expression cassette-dependent. In our study, the pcDNA3.1-based constructs consistently exhibited higher gD expression than the corresponding pCAG-based constructs when paired with the same signal peptide, as demonstrated by Western blot analysis, indicating that the CMV promoter in the pcDNA3.1 backbone was more effective than the CAG promoter for driving recombinant gD expression in CHO-K1 cells under the experimental conditions employed. These findings suggest that promoter selection plays a critical role in optimizing recombinant protein production, although the contribution of signal peptide choice should also be considered.

Within each vector backbone, the choice of signal peptide further modulated the amount of secreted protein. Irrespective of the vector used, the IL-2 signal peptide consistently outperformed both the GT6 and AP signal peptides in directing the secretion of the gD protein, as evidenced by the markedly higher band intensities quantified by grayscale densitometry of Western blot (Figure 1d). The IL-2 signal peptide is a well-characterized mammalian secretion signal derived from interleukin-2, a naturally secreted cytokine, and has been widely employed to enhance the secretory efficiency of recombinant proteins in mammalian expression systems [32]. Its superior performance relative to the GT6 (glycosyltransferase family 6) and AP (azurocidin preproprotein) signal peptides may be explained by more efficient co-translational recognition by the signal recognition particle (SRP) and more favorable translocation through the Sec61 translocon in the endoplasmic reticulum [33,34,35]. The combined effect of the strong CMV promoter-driven transcription and the efficient IL-2 signal peptide-mediated secretion resulted in the highest accumulation of recombinant gD protein in the culture medium, indicating that promoter activity and signal peptide efficiency act synergistically. This observation ultimately guided our choice of this construct for stable cell line establishment and subsequent scale-up.

The stable CHO suspension cell line generated in this study, adapted to serum-free culture conditions [29], provides a foundation for scalable and cost-effective manufacturing of the gD subunit vaccine, consistent with the growing application of CHO cells as the predominant platform for industrial production of recombinant biologics, including subunit vaccines [25]. For subsequent experiments, it would be valuable to investigate whether the expression levels can be further improved by employing engineered promoters with enhanced transcriptional activity, codon optimization strategies beyond those already applied, or the co-expression of molecular chaperones and folding modulators to facilitate ER-to-Golgi trafficking. Furthermore, evaluating the glycosylation profile of the gD protein produced by each construct would provide additional quality attributes relevant to immunogenicity, and differential signal peptide usage has been reported to influence N-glycan occupancy and processing.

The CHO-expressed gD subunit vaccine formulated with MONTANIDE ISA 206 adjuvant induced significantly higher neutralizing antibody titers compared with the commercial inactivated PRV vaccine (Ea strain) at 42 days post-immunization (*p* = 0.001). This superior neutralizing antibody response may be attributed to several factors. The CHO-expressed recombinant gD protein may harbor native-like post-translational modifications that preserve the conformational epitopes critical for inducing functional, virus-neutralizing antibodies; however, the glycosylation status of our recombinant gD protein was not experimentally characterized in the present study. In contrast, inactivated whole-virus vaccines contain a diverse mix of viral proteins, and can induce non-neutralizing antibodies that trigger immune effector functions, such as antibody-dependent cellular cytotoxicity. Our findings are consistent with prior studies demonstrating that gD-based vaccines elicit potent neutralizing antibody responses that correlate with protection [18,19,21,22,23].

Quantitative PCR analysis at 3 days post challenge revealed that viral genome copy numbers in the brain and spinal cord of vaccinated mice were reduced by more than 10^4^ copies per milligram of tissue compared with those in the PBS and adjuvant control groups (*p* < 0.0001). This remarkable reduction in CNS viral burden is particularly noteworthy given that the CNS is the primary target of PRV neuroinvasion. The protective efficacy of the gD subunit vaccine is consistent with the essential role of gD in mediating viral attachment and entry into host cells [15,16,17]; the fact that our gD-based vaccine nonetheless achieved complete protection against this highly neurotropic variant underscores the immunological relevance of the conserved neutralizing epitopes within gD.

The findings of the present study are broadly consistent with and extend the existing body of literature on gD-based PRV vaccines. Previous studies have shown that a baculovirus-expressed gD subunit vaccine elicited high-titer neutralizing antibodies in piglets that persisted for at least four months and provided complete protection against lethal challenge with the PRV-HNLH variant strain [18]. Similarly, researchers have reported that HEK-293T-expressed recombinant gD protein protected both mice and piglets against lethal PRV challenge and significantly reduced tissue viral loads and viral shedding [19]. Our results corroborate these findings and further demonstrate the utility of the CHO suspension cell platform—which is widely used for industrial-scale biopharmaceutical manufacturing [25]. Although experimental confirmation of the glycosylation profile of our gD protein remains to be performed, the CHO expression system offers the potential advantage of producing recombinant glycoproteins with mammalian-type glycosylation patterns that more closely mimic those of the native viral glycoprotein. Furthermore, previous studies have demonstrated that self-assembled LS-gD nanoparticles elicited robust humoral and cellular immune responses and provided effective protection in both mouse and piglet models, with no viral DNA detected in the brain and lung tissues of vaccinated mice [23]. While the level of CNS viral clearance achieved in our study is comparable to that reported for the nanoparticle vaccine [23], a direct head-to-head comparison would be necessary to determine the relative potency of these different gD-based vaccine modalities. Notably, our CHO-expressed monomeric gD subunit vaccine achieved profound CNS viral clearance without the need for nanoparticle display or multimerization strategies, suggesting that the structural integrity of the recombinant antigen, together with appropriate adjuvant formulation, may contribute to the induction of highly protective antibody responses.

In comparison with nucleic acid-based gD vaccines, previous studies have demonstrated that gD mRNA vaccines encapsulated in lipid nanoparticles elicited potent neutralizing antibody responses and complete protection in mouse models, with the additional advantage of stimulating robust CD4^+^ and CD8^+^ T-cell responses [21,22]. In the present study, we evaluated only humoral immune responses (gD-specific IgG and virus neutralization); cellular immune responses were not assessed, as detailed in the Limitations Section below. Our study did not evaluate cross-protection against heterologous PRV strains; the implications of gD sequence conservation for cross-protection are discussed below. Furthermore, previous studies on gD-based DNA vaccines have shown that the co-administration of STING agonists (cGAS, UniSTING, or IFN-*α*) significantly enhanced CD8^+^ T-cell responses and improved survival, further underscoring the importance of incorporating strategies to enhance cellular immunity in subunit vaccine design [20]. Additionally, researchers have demonstrated that Fc-fusion of gD (gD-IgG2aFc) enhanced both humoral and cellular immune responses compared with gD alone [24], suggesting that molecular adjuvant strategies could be applied to further improve the immunogenicity of our CHO-expressed gD antigen. Recently, a CHO-K1-derived gB and gD subunit vaccine was shown to provide complete clinical protection in piglets against variant PRV challenge [28]. Our study complements this work by providing a detailed description of the stable CHO suspension cell line development process—including signal peptide screening, clonal selection, and stepwise serum-free adaptation—for a gD-only subunit vaccine, and by offering quantitative evidence of profound viral clearance in CNS tissues.

The sequence alignment demonstrates that the gD extracellular domain is highly conserved among PRV strains circulating in China: the TJ strain-derived gD sequence shares 100% amino acid identity with the variant strains HeN1, HLJ8, WK1157, and WK631, and 99.75% identity with the classical SC strain. This high degree of conservation is consistent with the essential and non-redundant role of gD in mediating viral entry through interactions with conserved cellular receptors (nectin-1, nectin-2, and HVEM) [15,16], which likely imposes strong structural and functional constraints on the gD extracellular domain. These data, together with the cross-protection demonstrated by a gD mRNA-LNP vaccine against four genetically distinct PRV strains (HeN1, SC, TJ, and Bartha K61) [21], suggest that neutralizing antibodies induced by our CHO-expressed gD subunit vaccine may cross-react with other circulating variant strains, although this remains to be experimentally confirmed. Nevertheless, we acknowledge that sequence conservation alone does not constitute proof of functional cross-neutralization or cross-protection. Systematic cross-neutralization assays using immune sera against a panel of genetically diverse PRV isolates, as well as heterologous challenge experiments, will be required to experimentally define the breadth of protection conferred by this vaccine candidate.

Several limitations of the present study should be acknowledged. First, the immunogenicity and protective efficacy of the gD subunit vaccine were evaluated exclusively in a BALB/c mouse model. Although this model is widely used for the preclinical assessment of PRV vaccine candidates [19,21,22,23,24], mice are not the natural host of PRV, and several clinically important endpoints relevant to swine vaccination—including the prevention of virus shedding and transmission, protection against reproductive failure in pregnant sows, and the prevention of latent infection in trigeminal ganglia—cannot be assessed in mice. Therefore, the complete protection observed in the present study should be interpreted as a proof-of-concept that requires validation in the natural host. Second, only humoral immune responses (gD-specific IgG and virus-neutralizing antibody titers) were evaluated. Given that PRV is an *alphaherpesvirus* capable of establishing lifelong latent infection in sensory ganglia, cell-mediated immunity—particularly CD8^+^ cytotoxic T lymphocyte responses, CD4^+^ T helper cell activity, and IFN-*γ* production—is expected to play a critical role in clearing virus-infected cells and controlling viral reactivation [7,14]. The absence of cellular immune response data (e.g., IFN-*γ* ELISpot, intracellular cytokine staining, or lymphocyte proliferation assays) represents an important gap in the immunological characterization of our vaccine and should be addressed in future studies. Third, we did not experimentally characterize the glycosylation status of the purified recombinant gD protein. The contribution of CHO-specific glycosylation to the potent immunogenicity of herpesvirus glycoprotein subunit vaccines has been directly demonstrated for varicella-zoster virus, another member of the *Alphaherpesvirinae*: Nordén et al. showed that CHO-expressed recombinant VZV glycoprotein E (the antigen in the highly efficacious Shingrix^®^ vaccine) displays a characteristic O-glycosylation pattern that enhances the exposure and antibody recognition of key B cell epitopes compared with fibroblast-derived gE [36]. This precedent, together with evidence that CHO-expressed viral glycoproteins such as HIV-1 Env trimers can faithfully recapitulate native-like N-glycosylation at key neutralizing epitopes [37], supports the inference that CHO-specific post-translational modifications may contribute to the immunogenicity observed in our study. Nevertheless, experimental confirmation—by PNGase F digestion, lectin blotting, and/or LC-MS/MS-based site-specific N-glycan profiling—is essential and has been prioritized for future work. Fourth, viral replication was assessed solely by qPCR-based quantification of viral genome copy numbers, which does not distinguish between infectious and non-infectious viral particles. Infectious virus titration (TCID_50_ assay on tissue homogenates) and virus shedding measurements would provide complementary evidence that vaccination suppresses productive viral replication. Histopathological assessment (H&E staining) of brain and kidney tissues collected at 3 days post challenge showed no clear pathological differences between groups, likely owing to the acute nature of the challenge model and the early sampling time point relative to the development of overt tissue pathology [38,39]. In the present study, all control mice succumbed between 3 and 7 days post challenge—a time frame in which death is primarily attributable to acute neuroinvasion and hyperinflammatory responses rather than to established tissue-destructive pathology. Consequently, the profound reduction in CNS viral genome copy numbers (approximately 3.3–4.4 log_10_, Figure 6d) represents a more sensitive and informative endpoint than histopathology for quantifying vaccine-mediated suppression of viral replication in this acute challenge model. Future studies employing the natural swine host—in which the disease course is more protracted and classical histopathological lesions (neuronal degeneration, gliosis, and perivascular cuffing) have sufficient time to develop—would be better suited for histopathological evaluation of vaccine-induced protection against PRV-mediated tissue damage.

Beyond the limitations discussed above, several additional aspects warrant further investigation. First, the immunogenicity and protective efficacy of the CHO-expressed gD subunit vaccine should be evaluated in pigs, using clinically relevant challenge models that assess not only survival but also virus shedding, transmission dynamics, and prevention of latent infection in trigeminal ganglia [7,10,12,28]. Second, comprehensive cellular immune assays should be incorporated into future studies to establish the full immunological profile of the vaccine [20,21,22,23,24]. Third, the glycosylation profile of the CHO-expressed gD protein should be experimentally characterized [26,27]. Fourth, cross-protection against heterologous PRV variant strains should be systematically assessed [9,10,11,12,13,21]. Fifth, infectious virus titration and virus shedding measurements should be included as complementary virological endpoints. Sixth, the duration of protective immunity beyond the 42-day observation period needs to be determined; long-lasting immunity is a critical requirement for field vaccines, and Zhang et al. [18] demonstrated that gD-induced neutralizing antibodies can persist for at least four months, suggesting that durable immunity is achievable with gD-based vaccines. Seventh, the potential for dose-sparing, alternative adjuvant systems that promote balanced Th1/Th2 responses, and heterologous prime-boost regimens combining the CHO-expressed gD subunit vaccine with mRNA or viral-vectored vaccines should be explored to optimize both the magnitude and the breadth of protective immunity [20,21]. Eighth, the biosafety concern of genomic recombination between vaccine strains and field variant strains [11,13] further underscores the advantage of subunit vaccines over live attenuated vaccines, and this safety attribute of our CHO-expressed gD subunit vaccine should be emphasized in future development. Finally, process development studies aimed at increasing the volumetric productivity of the stable CHO-gD cell line, optimizing the purification workflow for large-scale manufacturing, and validating the long-term genetic stability of the producer cell line will be essential for translating this vaccine candidate from the laboratory to industrial production [25,29].

## 5. Conclusions

In conclusion, the gD subunit vaccine induced robust gD-specific and virus-neutralizing antibody responses and conferred complete protection against lethal PRV-TJ challenge in mice. These findings highlight the potential of CHO-derived gD as a PRV subunit vaccine antigen and support further evaluation in the natural swine host. However, its protective efficacy against PRV infection and transmission in pigs remains to be established.

## Data Availability

The datasets used and/or analyzed during the current study are available from the corresponding author upon reasonable request.

## Abbreviations

The following abbreviations are used in this manuscript:

PRV	Pseudorabies virus
PR	Pseudorabies
gD	Glycoprotein D
NAb	Neutralizing antibody
TCID_50_	50% Tissue culture infective dose
CHO	Chinese Hamster Ovary
SDS-PAGE	SDS-Polyacrylamide Gel Electrophoresis
qPCR	Quantitative Real-Time PCR
FBS	Fetal Bovine Serum
IgG	Immunoglobulin G
HRP	Horseradish Peroxidase
SP	Signal Peptide
dpc	Days post challenge
ANOVA	Analysis of variance
dpi	Days post immunization
